# Double‐row suture‐bridge technique does not yield better clinical and radiological results than single‐row technique in patients older than 55 years at 2 years minimum follow‐up: A comparative study

**DOI:** 10.1002/jeo2.70056

**Published:** 2025-05-05

**Authors:** Vito Gaetano Rinaldi, Sassoli Iacopo, Federico Coliva, Antongiulio Favero, Alberto Bazzocchi, Marco Miceli, Stefano Di Paolo, Stefano Zaffagnini, Giulio Maria Marcheggiani Muccioli

**Affiliations:** ^1^ II Clinica Ortopedica e Traumatologica—IRCCS Istituto Ortopedico Rizzoli Bologna Italy; ^2^ DIBINEM University of Bologna Bologna Italy; ^3^ Radiologia Diagnostica ed Interventistica—IRCCS Istituto Ortopedico Rizzoli Bologna Italy

**Keywords:** double‐row suture‐bridge, rotator cuff tears, shoulder arthroscopy, shoulder surgery, single row

## Abstract

**Purpose:**

Arthroscopic rotator cuff repair has evolved, with suture anchor‐based techniques like single‐row (SR) and Double‐row Suture‐bridge (DRSB) gaining popularity. Despite improvements, early repair failures remain concerning, necessitating continued assessment of repair methods and devices' lasting impact. This study compares DRSB versus SR repairs at 24 months minimum follow‐up, hypothesizing superior clinical outcomes and improved tendon healing with DRSB techniques.

**Methods:**

Fifty patients with rotator cuff tears underwent either SR or DRSB repairs. Clinical evaluation included standardized scoring systems and strength testing. Magnetic Resonance Imaging (MRI) assessed tendon integrity. Partial cuff tears were evaluated according to Snyder's Southern California Orthopaedic Institute rotator cuff classification system, which classifies <2 cm lesions as C2 in its scoring system.

**Results:**

Both groups showed comparable clinical outcomes, strength and MRI findings at 24 months minimum follow‐up. No significant correlation was found between repair technique and clinical outcomes or retear rates. Preoperative Patte and Goutallier grades >1 were associated with lower postoperative Constant–Murley scores.

**Conclusion:**

This study suggests that both SR and DRSB techniques offer comparable clinical outcomes and tendon healing rates for rotator cuff tears in patients over 55 at 24 months minimum follow‐up. While limitations exist, our findings contribute to understanding optimal surgical approaches, emphasizing individualized treatment based on patient characteristics and surgeon expertise. Further research, including randomized controlled trials with long‐term follow‐up, is needed to refine treatment algorithms and improve patient outcomes in rotator cuff surgery.

**Level of Evidence:**

Level III.

**Abbreviations**
DRSB: double‐row suture‐bridge
MRI: magnetic resonance imaging
RCR: arthroscopic rotator cuff repair
SMC: Samsung Medical Centre
SR: single‐row

## INTRODUCTION

During the last few years, arthroscopic approaches have progressively supplanted traditional open and mini‐open techniques for rotator cuff repair (RCR) [20]. Concurrently, suture anchor‐based fixation strategies for primary RCR have gained popularity due to their ease of use, superior biomechanical properties and various suture configuration options [7]. Both single‐row (SR) and double‐row suture‐bridge (DRSB) fixation techniques are prevalently employed to achieve a strong repair, exhibiting promising short‐term clinical outcomes [3, 9, 13, 17, 19, 32]. Despite these advances, early repair failures remain a concern, with retear rates fluctuating between 9% and 29% [4, 5, 27, 28].

Given the absence of a standardized approach for treating symptomatic rotator cuff tears (RCTs), there is still a continuous need for studies to assess the lasting impact of contemporary repair methods and employed devices. According to several studies, nearly half of the patients experience recurrent tears following arthroscopic SR repairs [30, 33, 34, 37, 38]. Indeed, these repair failures are not merely academic concerns; they are linked to functional limitations and the progression of osteoarthritic changes.

At the state of the art, there is still no universally accepted standard for repair methodology. Current studies compare SR and DR constructs to identify the optimal approach. Recent evidence suggests that DR repair may provide a biomechanically superior construct, albeit at a higher cost and time investment than SR repair [12]. Surgeons primarily rely on personal surgical experience to choose between SR and DR methods. Only <2 cm lesions were chosen to be repaired. The surgeon began with the simplest SR technique and, as he progressed along the learning curve, he also performed DR repairs. As it stands in contemporary literature, the DR repair technique should be chosen to gain early functional recovery in high‐demand patients.

Over the years, all the articles published have analyzed clinical outcomes, mainly using scoring systems such as the American Shoulder and Elbow Surgeons (ASES) score [22], the University of California‐Los Angeles (UCLA) [36] and the Constant–Murley score [8], with results that are often inconsistent with each other [2].

Furthermore, not all articles in the existing literature comprehensively analyze the potential for retearing the repaired rotator cuff. There is a significant inconsistency in the methods used for assessing tendon integrity. While some authors have relied on magnetic resonance imaging (MRI) scans at a maximum power of 1.5 T for their evaluations [33, 37], others have used ultrasound exclusively [16, 19, 39].

Remarkably, no studies have explored the integrity of tendon repair using a 3 T MRI.

The present study compares the efficacy of DRSB versus SR anchor repairs in rotator cuff arthroscopic surgery. To our knowledge, no previous studies are available confronting these two repair techniques. It was hypothesized that DRSB repair techniques would yield superior clinical outcomes, as measured by standardized scoring systems, and would demonstrate improved tendon healing, as assessed by 3‐T MRI, compared to their SR counterparts.

## METHODS

This study received ethical approval from the appropriate institutional review board prior to the commencement (number: 805‐2022).

Fifty patients were included in this retrospective study conducted from January 2021 to December 2023 at IRCCS Istituto Ortopedico Rizzoli, Bologna, Italy. All participating patients provided informed consent for their data to be included in the study. Eligibility criteria were as follows: full‐thickness tear of a single posterosuperior rotator cuff tendon confirmed by MRI and subsequent surgery, a willingness to adhere to a standardized rotator cuff physical therapy programme and a tear pattern amenable to either SR or DR fixation techniques, as determined during surgery.

Patients were excluded from the study if they had an active smoking history, autoimmune or rheumatologic diseases, active steroid usage, prior rotator cuff surgery on the affected shoulder or a tear pattern at the time of surgery deemed unrepairable with the SR or DRSB technique employed in this study. Additionally, patients affected by subscapularis, traumatic and massive RCTs were excluded.

Moreover, all patients were classified according to the Goutallier [11], Patte and Snyder's [1, 31] classifications; tears >2 Patte grade, >1 Goutallier and Snyder's aside from C2 grade were excluded from the study.

All the patients underwent surgery between 2015 and 2020.

A power analysis was conducted using the G*Power software, and it was determined that each group needed at least 25 patients to achieve statistical significance.

Participants were split into two equal groups of 25 patients, differentiated by the repair technique. Group 1 consisted of patients who underwent an arthroscopic RCR using the SR technique. In contrast, Group 2 included patients treated with the DR suture‐bridge technique.

This was a retrospective study, so a priori randomization was impossible. Instead, data from patients who underwent two different rotator cuff suture techniques were analyzed retrospectively and then prospectively. All procedures were performed by the same surgeon (G. M. M. M.).

The choice of repair type (SR vs. DRSB) was purely random and was mainly influenced by the surgeon's learning curve. The initial patients were primarily treated with the SR technique, while more recent patients were treated with DRSB.

### Surgical technique

All surgical interventions were conducted with patients in a beach chair position and under general anaesthesia. A standard arthroscopic pump and routine portals were utilized for the procedures. After performing a standard intra‐articular examination, the arthroscope was inserted into the subacromial space via the posterior portal, followed by the creation of a lateral portal. Complete bursectomy and arthroscopic subacromial cleaning were performed to achieve a better view. If necessary (rarely), osteophytes in the inferior part of the acromioclavicular joint were also excised.

Subacromial decompression through acromioplasty has never been performed in accordance with the surgeon's preferred surgical technique. Tenotomy or tenodesis of the long head of the biceps was performed only in cases of evident tendon injury or of the tendon pulley, depending on the patient's characteristics, functional request and age.

The size of each RCT was quantitatively assessed arthroscopically at the time of surgery using a probe. The probe served as a ruler for measuring the tear size, categorizing tears smaller or equal to 2 cm^2^ as small‐to‐medium tears. The study explicitly excluded patients with large or massive tears, L‐shaped tears, as well as those exhibiting combined intra‐articular pathologies such as superior labrum anteroposterior, Bankart or chondral lesions.

For the SR repair, anchors were strategically positioned at the articular margin of the humeral head's superior face. The number of anchors used ranged from one to two, depending on the tear size. Corkscrew 5.5 mm metallic anchors preloaded with No. 2 Fiberwire sutures (Arthrex) were employed for the procedure. Once the anchors were inserted via the superior portal, simple mattress stitches were individually tied from these double‐loaded anchors into the lateral edge of the torn tendon. The sutures were placed at 10–15 mm of tendon tissue using a Scorpion Suture Passer (Arthrex).

**Figure 1:**
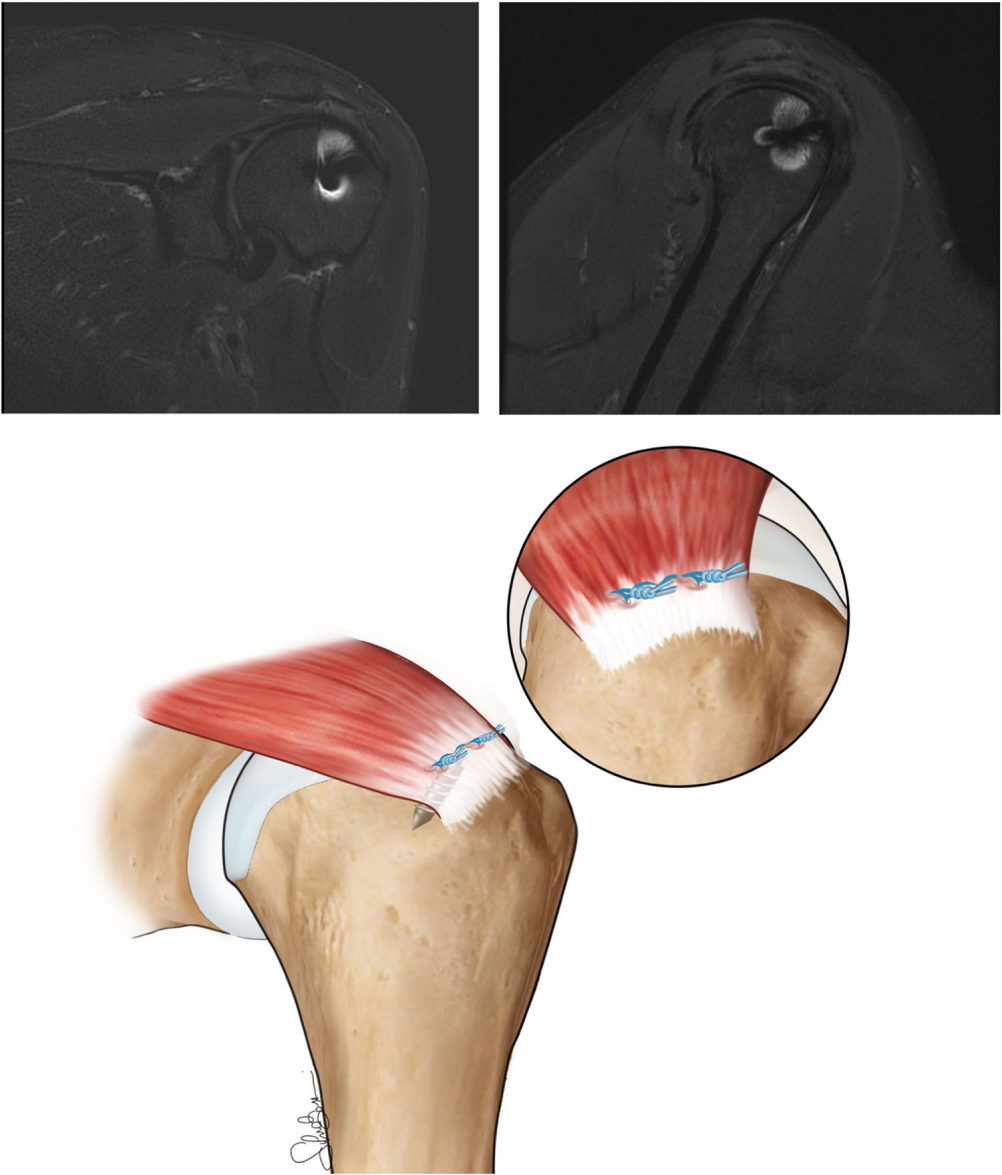
Single‐row technique at 24 months minimum follow‐up. 3‐T MRI images show the supraspinatus tendon reattached to its footprint using one Corkscrew 5.5 mm metallic anchors preloaded with No. 2 Fiberwire sutures (Arthrex). The tendon appears continuous and well‐integrated. MRI, magnetic resonance imaging.

After the sutures were positioned, they were sequentially secured using a locking, sliding Samsung Medical Centre knot supplemented by five backup half‐hitches, switching the post each time (Figure 1).

**Figure 2:**
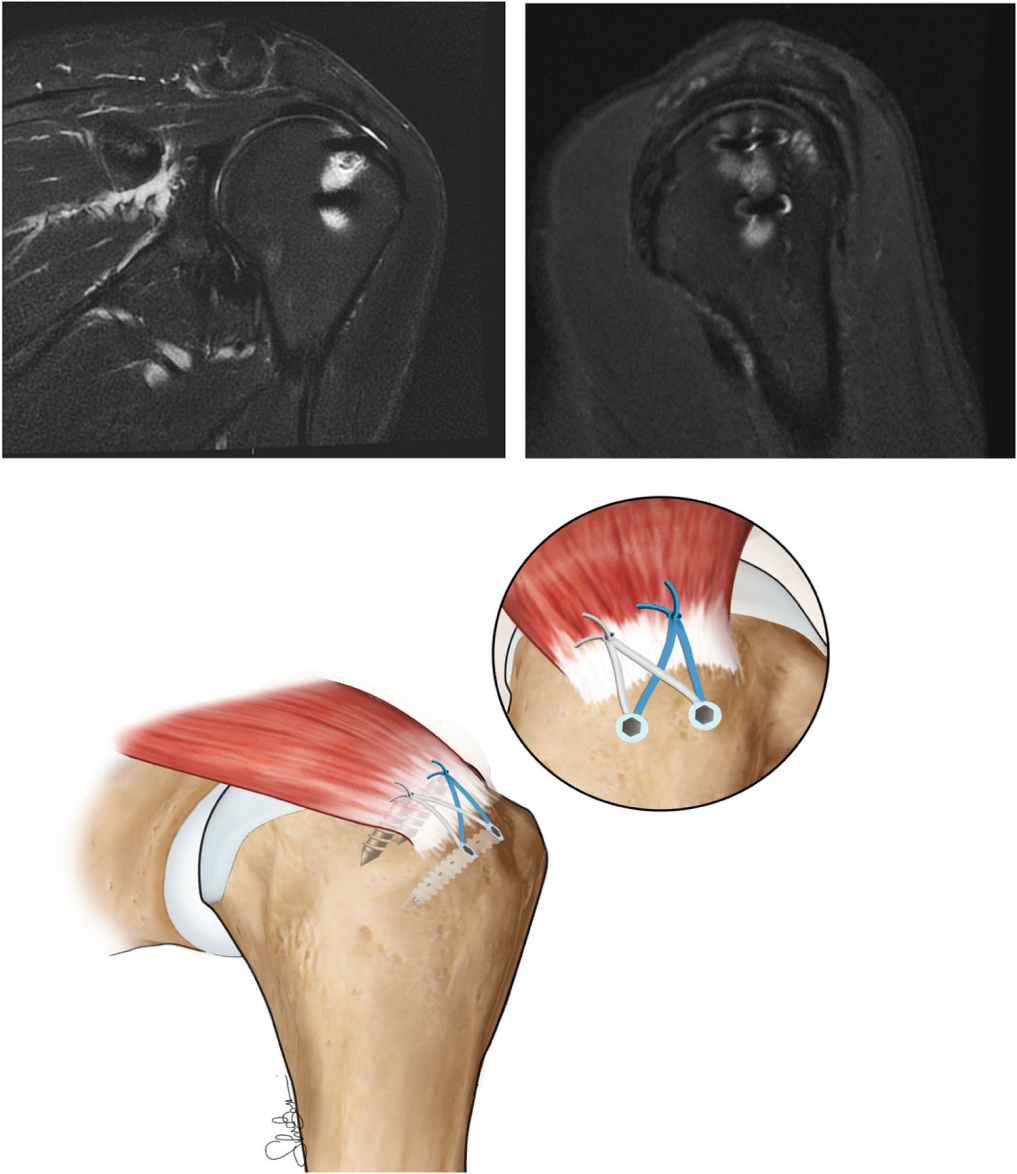
Double‐row suture‐bridge technique at 24 months minimum follow‐up. 3‐T MRI images show the supraspinatus tendon reattached to its footprint using two Corkscrew 5.5 mm metallic anchors preloaded with No. 2 Fiberwire sutures (Arthrex) and two footprint knotless anchors (SwiveLock SP 4.75 mm, Arthrex). The tendon appears continuous and well‐integrated. MRI, magnetic resonance imaging.

In the case of DRSB repair, the medial row consisted of one or two anchors placed at the articular margin of the humeral head. Both limbs of each suture were threaded through the tendon, approximately 15 mm medial to the tear margin. The lateral row of anchors was situated on the lateral edge of the native tendon footprint. The number of anchors, again, varied between one to two based on the tear size. One or two footprint knotless anchors (SwiveLock SP 4.75 mm, Arthrex) were used for the lateral row, loaded with sutures from the medial row, thereby creating a suture bridge between the medial and lateral rows (Figure 2).

Postoperatively, patients were fitted with a shoulder‐immobilizing sling with a 20° abduction pillow. Gentle passive forward flexion closed chain table‐slide exercises were initiated the day after surgery. The duration of immobilization with the abduction pillow depended on factors such as tear size, tissue quality and the robustness of the repair. Patients could only remove their arms from the sling during exercise. Active‐assisted range of motion (ROM) exercises typically commenced 4–6 weeks postoperatively, transitioning to full active ROM between 6 and 8 weeks.

### Clinical evaluation and strength evaluation

Physical examinations were conducted for all the patients. Functional assessments were made using the Constant–Murley shoulder scoring system [8], the ASES score [29] and the UCLA score [21] at 24 months minimum of follow‐up.

Clinical evaluations were conducted by a skilled shoulder surgeon (V. G. R.), while the second researcher (I. S.) performed strength testing.

**Figure 3:**
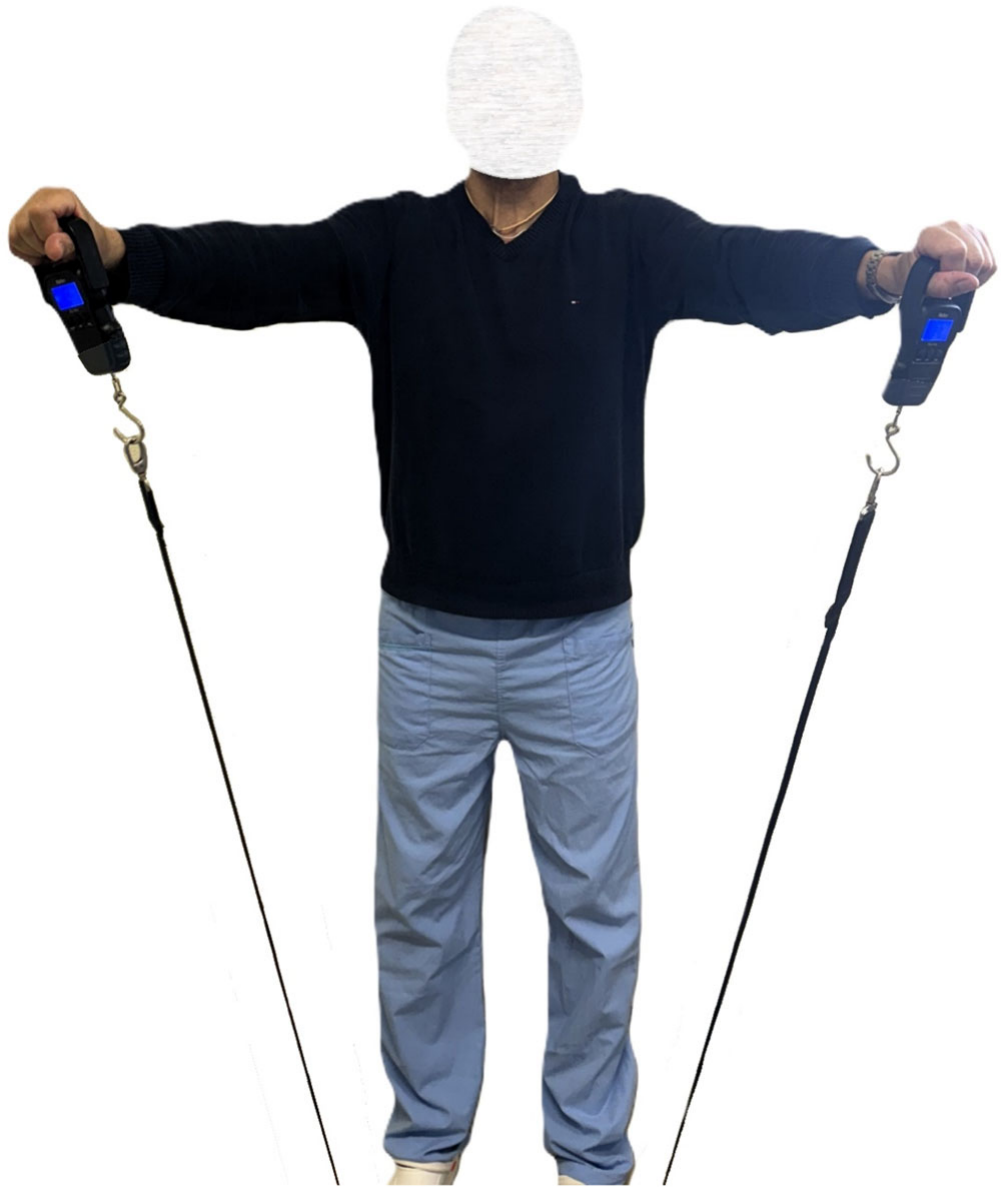
Strenght evaluation during the Jobe test using dynamometers: The patient holds below his feet the mechanical resistance while the observer evaluates quantitative strength, by reading the results on the display. Arms are kept on the scapular plane and the patient is asked to uplift his limbs three consecutive times. Values are annotated.

**Figure 4:**
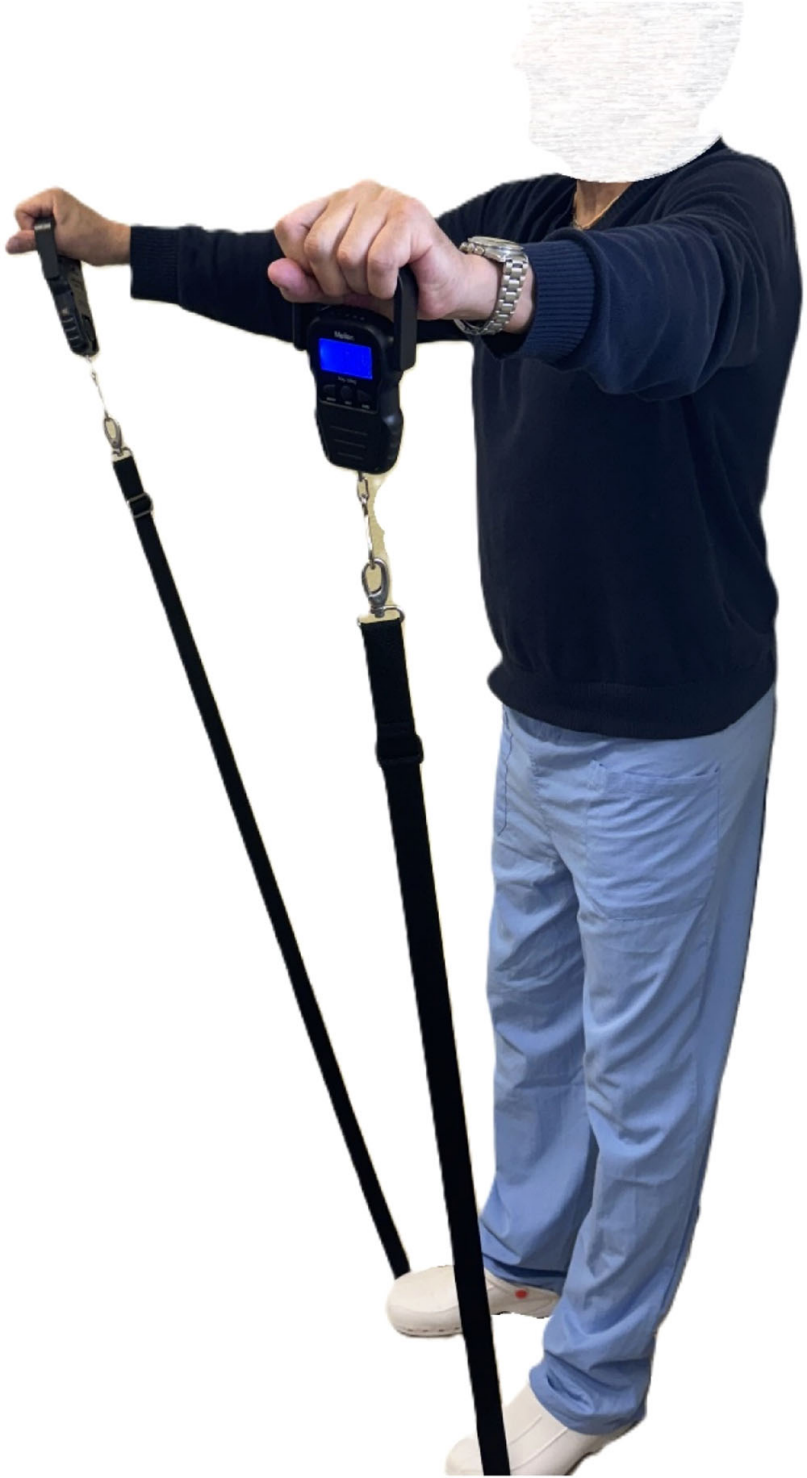
Lateral view.

Strength evaluation at the Jobe test [15] was performed. The patient was standing with his back adherent to a wall and the arms were placed in a starting position of 90° of abduction, 30° of forward flexion and with the elbow extended (in the scapular plane). Two dynamometers (Meilen, MS003Y‐g) were held by the patient on each hand and fixed under the patient's feet using two strings (Figures 3 and 4). The Jobe test was performed three times, and the best pull was recorded for both the operated and the nonoperated sides. Maximum strength (expressed in kilograms) and the differential strength (nonoperated—operated side strength) were reported in our data collection.

### MRI and ‐rotator cuff integrity assessment

MRI was performed before (1–3 months) and at minimum 2 years after surgery, using a 3 T MR scanner (Discovery MR750w GEM, GE Healthcare). MRI protocol included oblique coronal T2w, oblique sagittal T2w and axial T2w, both preoperatively and postoperatively.

Two musculoskeletal radiologists, with more than 15 (A. B.) and 25 (M. M.) years of experience, respectively, evaluated all MR exams in consensus and blinded to surgical and clinical background and outcomes.

In order to assess interobserver reliability, the Intraclass Correlation Coefficient (ICC) was calculated to compare the results between two observers.

Prior to surgery, structural and qualitative assessment of the rotator cuff was provided, and Goutallier [11] and Patte [31] classification was determined for each patient.

Postoperatively, rotator cuff integrity was scored according to Sugaya classification [35] (Table 1).

**Table 1:** Rotator cuff integrity classified according to Sugaya et al.

Type 1	Repaired cuff with sufficient thickness compared with normal cuff, and homogenously low signal intensity on each image.
Type 2	Repaired cuff with sufficient thickness compared with normal cuff, associated with partial high signal intensity area.
Type 3	Repaired cuff with insufficient thickness with less than half the thickness when compared with normal cuff, but without discontinuity, suggesting a partial‐thickness delaminated tear.
Type 4	Presence of a minor discontinuity in only one or two slices on both oblique coronal and sagittal images, suggesting a small full‐thickness tear.
Type 5	Presence of a major discontinuity observed in more than two slices on both oblique coronal and sagittal images, suggesting a medium or large full‐thickness tear.

### Statistical analysis

Statistical analysis was executed using SPSS 11.0 software.

Data distribution was inspected through the Shapiro–Wilk test. Normally distributed data were presented as mean ± standard deviation; nonnormally distributed and categorical data were presented as median ± interquartile range and percentage over the total.

The *t* test was employed to compare preoperative and postoperative Constant scores between the two groups.

Multiple regression analyses were performed, considering Patte, Gouttalier, age and surgical technique as independent variables and ASES, UCLA and Constant as dependent variables.

Multinomial logistic regression analyses were also performed using the same independent variables with Sugaya, Delta force and Constant with respective cut‐offs (SUGAYA > 2, DELTA > 0, CONSTANT > 85, respectively) as dependent variables. Adjusted *R* ^2^, standardized and unstandardized coefficient and *p* values were reported for the three linear regressions. Cox *R* ^2^, Odds ratio and *p* values were reported as outcomes for logistic regressions. Differences were considered statistically significant for *p* < 0.05. All analyses were performed in SPSS.

To ensure the statistical robustness of our study, a power analysis using G‐power software (version X) was conducted. Specifically, it was aimed for a power of 0.80, an alpha level of 0.05 and an effect size based on previous literature in the field.

## RESULTS

The mean follow‐up was 56 ± 10 months. Twenty‐five patients were treated for an isolated supraspinatus tendon lesion with either an SR or a DRSB arthroscopic repair. The mean preoperative Patte grade was 1.84, and the Goutallier grade was 0.5. The demographics of our study population are shown in Table 2. No statistically significant difference was found between the two groups.

**Table 2:** Patients' demographics and characteristics.

	Overall	SR	DRSB
**Number of patients**	50	25	25
**Age**	58.06 (27–74) ± 9.75	54.6 (27–71) ± 10.90	61.52 (43–74) ± 7.08
**Gender**	32 M/18 W	15 M/10 W	17 M/8 W
**Patte**	1.84 (1–4) ± 0.79	1.72 (1–4) ± 0.84	1.96 (1–3) ± 0.73
**Goutallier**	0.5 (0–2) ± 0.54	0.36 (0–1) ± 0.49	0.64 (0–2) ± 0.57

*Note*: Age: reported in years (range) standard deviation; Patte: mean preoperative tendon retraction according to Patte Classification (range) standard deviation; Goutallier: mean preoperative tendon fatty infiltration according to Goutallier Classification (range) standard deviation.

Abbreviations: DRSB, double‐row suture‐bridge repair technique; M, men; SR: single‐row repair technique; W, women.

The clinical results are shown in Table 3. The SR group had a mean postoperative value at ASES, UCLA and Constant–Murley score of 84 (range 37–100, standard deviation 17.61), 27.6 (range 12–35, standard deviation 7.44) and 93.64 (range 76–100, standard deviation 6.20), respectively, at the final follow‐up. Absolute strength at the Jobe test was found to be 5.74 kg (range 2.04–8.84 kg, standard deviation 1.58), and the mean strength difference with the nonoperated side was −1.72 kg (range 7.44–4.59 kg, standard deviation 3.10).

**Table 3:** Clinical and radiological outcomes of different surgical techniques.

	Overall	SR	DRSB
**ASES**	86.82 (37–100) ± 15.60	84 (37–100) ± 17.60	89.72 (57–100) ± 13.01
**UCLA**	28.68 (12–35) ± 6.62	27.6 (12–35) ± 7.44	29.76 (18–35) ± 5.64
**CM**	92.96 (70–100) ± 6.47	93.64 (76–100) ± 6.20	92.28 (70–100) ± 6.79
**Strength at jobe**	6.20 (1.67–14.93) ± 2.38	5.74 (2.04–8.84) ± 1.58	6.66 (1.67–14.93) ± 2.93
**Δ Strength**	−1.77 (−7.7–5) ± 2.88	–1.72 (−7.44–4.59) ± 3.10	–1.81 (−7.7–5) ± 2.68
**Sugaya**	2.04 (1–5) ± 0.90	1.92 (1–4) ± 0.91	2.16 (1–5) ± 0.90
**Goutallier**	1.04 (0–3) ± 0.88	0.88 (0–3) ± 0.93	1.2 (0–3) ± 0.82

*Note*: ASES: mean (range) standard deviation; UCLA shoulder score: mean (range) standard deviation; CM shoulder score mean (range) standard deviation; Strength at Jobe: strength measured with dynamometer at Jobe test expressed in kilogram mean (range) standard deviation; Strength: differential strength between operated and nonoperated side expressed in kilogram mean (range) standard deviation; Sugaya: Retear rate at final follow‐up MRI according to Sugaya classification mean (range) standard deviation; Goutallier: mean postoperative tendon fatty infiltration according to Goutallier Classification (range) standard deviation.

Abbreviations: ASES, American Shoulder and Elbow Surgeons score; CM, Constant–Murley shoulder score; DRSB, double‐row suture‐bridge repair technique; MRI, magnetic resonance imaging; SR, single‐row repair technique; UCLA, University of California Los Angeles shoulder score.

The DRSB group showed a mean postoperative ASES value of 89.72 (range 57–100, standard deviation 13.01), a mean UCLA value of 29.76 (range 18–35, standard deviation 5.64) and a mean Constant–Murley score of 92.28 (range 70–100, standard deviation 6.79). The dynamometer measured strength at the Jobe test was found to be 6.66 kg (range 1.67–14.93 kg, standard deviation 2.93) with a measured mean difference with the nonoperated side of −1.81 kg (range 7.7–5 kg). Three shoulders were excluded from the contralateral side difference evaluation for the presence of a diagnosed rotator cuff lesion on the nonoperated shoulder.

The radiological results of the Sugaya score at the final follow‐up had a mean value of 1.92 (range 1–4, standard deviation 0.91) for the SR group and 2.16 (range 1–5, standard deviation 0.90) for the DRSB group; the Goutallier grade were 0.88 (range 0–3, standard deviation 0.93) and 1.2 (range 0–3, standard deviation 0.82), respectively.

The ICC results demonstrated a high degree of agreement between the two observers, with an ICC value of 0.92 (95% confidence interval: 0.88–0.96), indicating consistent and comparable evaluations.

In Table 4, the correlation analysis of our work is summarized. No statistically significant correlation between the surgical technique and higher postoperative Constant–Murley, ASES or UCLA scores nor with comparative strength at the Jobe test or the Sugaya retear rate was found.

**Table 4:** Statistical correlation.

	ASES	UCLA	CM	Sugaya > 2	Strenght > 0
Age	0.265	0.348	0.75	0.59	0.974
Patte > 1	0.38	0.418	***0.217*** *	0.069	0.598
Goutallier > 0	0.442	0.424	***0.027*** *	0.245	0.306
Surg.tech (SR)	0.152	0.234	0.77	0.616	0.479

*Note*: Sugaya: Retear rate at final follow‐up MRI according to Sugaya classification >2; Strength: differential strength between operated and nonoperated side expressed in kilogram >0; Patte: mean preoperative tendon retraction according to Patte Classification >1; Goutallier: mean preoperative tendon fatty infiltration according to Goutallier Classification >0; Surg.Tech(SR): between surgical technique used (single‐row repair) and clinical results reported.

Abbreviations: ASES, American Shoulder and Elbow Surgeons score; CM, Constant–Murley shoulder score; MRI, magnetic resonance imaging; UCLA, University of California Los Angeles shoulder score.

* Statistically significant correlation.

The population was also divided based on preoperative Patte, Goutallier and age. It was analyzed whether there was a statistically significant correlation between our clinical and radiological outcomes. Our series showed that preoperative Patte and Goutallier grades >1 were statistically correlated with a lower Constant–Murley clinical score in a linear regression model.

## DISCUSSION

The primary finding of our study highlights that no significant differences were observed in our population at 24 months minimum follow‐up between the SR and DRSB techniques in terms of clinical outcomes, strength measured by dynamometers at the Jobe test and 3‐T MRI assessments when utilized for treating RCTs.

In addition to our findings, it is important to contextualize our results within the broader body of literature on RCR techniques. Numerous studies have investigated the comparative effectiveness of SR and DRSB techniques, yielding miscellaneous results.

In our study, the lack of significant differences in clinical outcomes suggests that both SR and DRSB techniques are effective in treating small‐to‐medium size (≤2 cm) RCTs in patients older than 55 years of age. This finding aligns with previous studies that have also failed to demonstrate the superiority of one technique over the other in similar patient populations [6, 10, 12, 14, 19, 23, 24, 30, 34, 37].

For instance, a recent meta‐analysis by Lapner et al. [18] concluded no significant difference in clinical outcomes between SR and DR repairs for small to medium‐sized RCTs.

Moreover, several authors have demonstrated that there is no difference in terms of clinical outcomes between SR and DRSB techniques.

In particular, Mihata et al. [23], Grasso et al. [12] and Charousset et al. [6] have provided further evidence supporting the lack of significant differences in clinical outcomes between SR and DR techniques for RCR.

In contrast, the outcomes of a recent systematic review of 13 randomized trials shed light on the advantages of the DR technique in RCR. Notably, superior UCLA scores, enhanced tendon healing rates and decreased retear rates were observed, affirming the efficacy of this approach. However, intriguingly, the same authors found no discernible clinical disparities in either the Constant–Murley or the ASES score [26]. This nuanced revelation challenges prevailing assumptions and highlights the complexity of evaluating surgical outcomes in RCR.

Furthermore, our evaluation of strength using dynamometers during the Jobe test showed comparable results between the two techniques, revealing a lack of superiority of one technique over the other in terms of measured strength. This suggests that both approaches can similarly restore muscle function and strength after RCT repair in low‐demand patients (age >55) with medium‐to‐small tear sizes.

These findings are in contrast with previous studies that have investigated muscle strength following RCR using different suture techniques. For example, Milano et al. [24] conducted a biomechanical in vitro study comparing strength between SR and DR techniques, finding significant differences between the two groups. Additionally, another biomechanical study by Wall et al. [38] reached similar conclusions, highlighting the superiority of the DR technique.

These discordant results may be attributed to these studies from approximately 15 years ago, where the techniques and, most importantly, the materials utilized are outdated. In our cohort of patients, modern anchors were used along with the most innovative suture techniques and settings.

To the best of the authors’ knowledge, this is the first study to evaluate the strength by dynamometers following RCR at a minimum 24‐month follow‐up.

Radiologically, our assessment using 3‐T MRI and Sugaya score did not reveal any notable superiority between the two techniques. This suggests that both methods achieve satisfactory structural integrity and healing of the repaired tendon, as evidenced by similar MRI findings. This is in accordance with the findings of a systematic review by Hein et al. [14], which found no significant difference in structural outcomes between SR and DRSB repairs.

Also, Koh et al. [17] are in line with the statement mentioned above, suggesting that the radiological outcomes and retear rates of DRSB repair were not significantly different from those of SR repairs in patients with medium‐to‐small RCTs.

Ning et al., in contrast, demonstrated significantly superior healing with the DR suture‐bridge technique compared to the SR technique at 1.5 T MRI evaluation using the Sugaya classification 12 months postsurgery in moderate RCTs [25].

Importantly, to the best of our knowledge, no study has been conducted to date evaluating patients with MRI at 3 T, making our study unique in this field.

The limitations of this study include the retrospective design, which inherently introduces potential biases and limitations in data collection and analysis. Additionally, the lack of randomization in patient allocation to the SR and DR repair groups may have led to selection bias and confounding variables that could influence the study outcomes. Moreover, the study's sample size of fifty patients may not be sufficient to detect small but clinically significant differences between the two repair techniques, particularly in subgroup analyses or for rare outcomes such as retear rates.

Another limitation is the reliance on subjective clinical assessment tools, such as the Constant–Murley shoulder scoring system, the ASES score and the UCLA score, which may introduce variability and subjectivity in evaluating patient outcomes. Additionally, the lack of standardized outcome measures for assessing tendon integrity and healing, particularly the absence of 3‐T MRI evaluation in the existing literature, limits the comparability and generalizability of the study findings.

Moreover, the study did not evaluate long‐term outcomes beyond the minimum 24‐month follow‐up period, which may fail to capture late complications.

In conclusion, this study contributes to the ongoing discussion regarding the optimal surgical approach for treating RCTs. Despite the limitations outlined, our findings suggest that both SR and DRSB repair techniques exhibit comparable clinical outcomes and tendon healing rates for RCTs. These results are consistent with previous literature, highlighting the lack of significant differences between the two techniques in similar patient populations.

However, it is essential to acknowledge the evolving landscape of surgical techniques and technological advancements in RCR. Emerging approaches, such as knotless suture anchor fixation and augmented repair techniques, may influence the comparative effectiveness of SR versus DR repairs. Future studies should explore these innovative techniques and their potential impact on clinical outcomes and long‐term tendon healing.

Furthermore, selecting an optimal surgical technique should be individualized based on patient and lesion characteristics, surgeon expertise and available evidence. While single‐ and DR repairs offer viable options for RCR, carefully considering patient‐specific factors and surgical goals is essential in determining the most appropriate approach.

## CONCLUSIONS

In conclusion, while our study provides valuable insights into the comparative effectiveness of SR and DR repair techniques, further research is needed to refine treatment algorithms and improve patient outcomes in rotator cuff surgery. Randomized controlled trials with standardized outcome measures and long‐term follow‐up are warranted to address the remaining uncertainties and optimize the management of RCTs.

## AUTHOR CONTRIBUTIONS

All authors have contributed to the development of the research questions and study design. Vito Gaetano Rinaldi and Giulio Maria Marcheggiani Muccioli identified the method of the study. Giulio Maria Marcheggiani Muccioli performed the surgeries. Alberto Bazzocchi and Marco Miceli performed the radiological evaluation. Vito Gaetano Rinaldi, Sassoli Iacopo, Federico Coliva and Antongiulio Favero developed and conducted the follow‐up. Stefano Di Paolo performed the statistical analysis. Vito Gaetano Rinaldi, Sassoli Iacopo, Federico Coliva and Antongiulio Favero developed the first and subsequent drafts of the manuscript and Stefano Zaffagnini revised it. All authors reviewed and approved the manuscript.

## CONFLICT OF INTEREST STATEMENT

The authors declare no conflict of interest.

## ETHICS STATEMENT

This study received approval from our institution ethics committee. All participants provided written informed consent before participating in the study. Participants provided consent for the publication of anonymized data and any accompanying images. Personal identifiers have been removed in the manuscript to ensure confidentiality and protect the privacy of the participants.

## Supporting information

Supporting information.

Supporting information.

## Data Availability

The data sets used and analyzed during the current study are available from the corresponding author.
